# Effect of Material Extrusion Process Parameters to Enhance Water Vapour Adsorption Capacity of PLA/Wood Composite Printed Parts

**DOI:** 10.3390/polym16202934

**Published:** 2024-10-19

**Authors:** José A. Martínez-Sánchez, Pablo E. Romero, Francisco Comino, Esther Molero, Manuel Ruiz de Adana

**Affiliations:** 1 Departamento de Mecánica, Escuela Politécnica Superior, Universidad de Córdoba, Campus de Rabanales, Antigua Carretera Nacional IV, km 396, 14071 Córdoba, Spain; z12masaj@uco.es (J.A.M.-S.); francisco.comino@uco.es (F.C.); esther.molero@uco.es (E.M.); 2 Departamento de Química-Física y Termodinámica Aplicada, Escuela Politécnica Superior, Universidad de Córdoba, Campus de Rabanales, Antigua Carretera Nacional IV, km 396, 14071 Córdoba, Spain; manuel.ruiz@uco.es

**Keywords:** polylactic acid (PLA), wood, material extrusion, desiccant materials, water adsorption

## Abstract

This study aims to optimise the water vapour adsorption capacity of polylactic acid (PLA) and wood composite materials for application in dehumidification systems through material extrusion additive manufacturing. By analysing key process parameters, including nozzle diameter, layer height, and temperature, the research evaluates their impact on the porosity and adsorption performance of the composite. Additionally, the influence of different infill densities on moisture absorption is investigated. The results show that increasing wood content significantly enhances water vapour adsorption, with nozzle diameter and layer height identified as the most critical factors. These findings confirm that composite materials, especially those with higher wood content and optimised printing parameters, offer promising solutions for improving dehumidification efficiency. Potential applications include heating, ventilation, and air conditioning systems or environmental control. This work introduces an innovative approach to using composite materials in desiccant-based dehumidification and provides a solid foundation for future research. Further studies could focus on optimising material formulations and scaling this approach for broader industrial applications.

## 1. Introduction

Material Extrusion (MEX) is an additive manufacturing (AM) process that allows the production of parts or components layer by layer by melting and depositing a thermoplastic-based filament [1]. MEX is a very popular technology due to several reasons [2,3,4]: it enables the production of complex, functional parts on demand in a single step; it allows for multi-material fabrication; the equipment is cost-effective, easy to operate, and maintain; it supports the production of large-format parts; and it offers numerous adjustable printing parameters to suit various applications.

**Figure 1:**
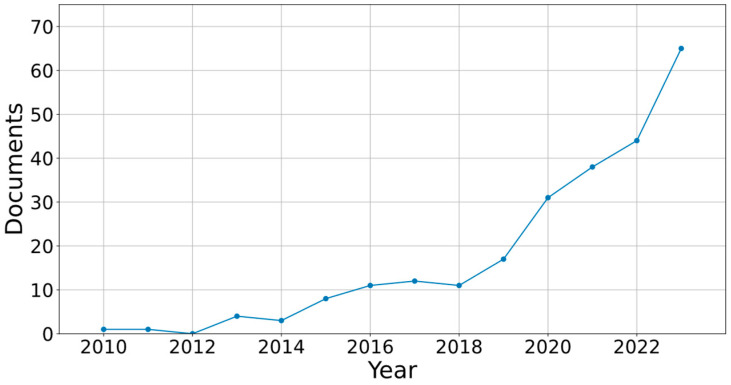
Documents published from 2010 to 2023 when searching Scopus for the keywords “Additive manufacturing” and “Wood”.

The demand for and research into Multi-Material Additive Manufacturing (MMAM) is continually growing [5,6,7], and the catalogue of materials is increasing year by year, thanks to the work of companies, universities, and research centres [8,9,10]. In this context, renewable organic polymers are widely used in MEX technology and combined with synthetic materials to achieve the desired printability and features of 3D printed structures [11]. Some of these composites are collagen and polymer composites, or graphene architectures [12,13]. Polylactic acid (PLA)/Wood is one of the most promising materials [14]. In Figure 1, the interest in wood-based materials used in this technology can be observed. PLA/wood is known as wood polymer composite (WPC) [15], and it has several benefits: it is easy to print, thanks to PLA [16]; it also allows the production of lighter parts (the density of wood is lower than that of PLA), although rougher and with a higher number of pores [17]; and PLA/wood has an organic origin and is biodegradable [18], a fact that contributes to generating a circular economy [19,20]. Some authors have studied the recyclability of emergent biocomposites [21] as well as wood polymer composites (WPCs). Although recycling composite materials presents challenges due to the combination of materials, recent studies have demonstrated effective reuse solutions. Copenhaver et al. [22] showed that wood polymer composite (WPC) components can be shredded and reused via 3D printing or injection molding to create new parts. Ngaowthong et al. [23] also highlighted that incorporating agricultural waste into polymer composites improves their recyclability. Additionally, the environmental impact of PLA/wood parts printed via 3D printing is considerably lower than that of similar metal components. At the end of its life cycle, PLA is recyclable without quality degradation [24], making it a sustainable choice compared to conventional materials. Industrial composting, recycling, and reuse represent key solutions to reduce the ecological footprint of these composites. Previous studies have shown that PLA/wood has a higher porosity than virgin PLA, leading to reduced cross-sectional strength and increased stiffness [25,26].

Several authors have analysed the water absorption capacity of PLA/wood composite materials [27,28]. Water sorption in solid materials can be identified in four phases: hydration water, surface water, interstitial water, and free water. Each phase has distinct characteristics and retention mechanisms within the material [29]. Hydration water, or intracellular water, is chemically bonded within the material, exhibiting the strongest bonding force among the phases. Surface water is bound through hydrogen bonds, adsorption, and adhesion or chemical adsorption. The thickness of the water layer is approximately 3–5 nm. Interstitial water is retained by capillary forces within the material. Free water does not exhibit any binding force and is the easiest to remove. The first two types of water, hydration water and surface water, can only be removed through the application of thermal energy [29].

Recent research works found that absorption capacity increased in materials with high surface roughness and porosity, and discovered how the mechanical properties of the material vary when water sorption occurs, showing that these properties can be reduced [30]. For this reason, some authors have studied how to reduce this affinity for water. Elumalai et al. has verified that the use of epoxy coating like Araldite LY556 reduced the water absorption by 89.96% [31]. On the other hand, Shulga et al. have verified that the incorporation of lubricants in the composite formulation leads to a decrease in the water absorption [32]. Krapez-Tomec et al. have shown that the use of thermally modified wood decreases water absorption under humid conditions [33]. Segerhom et al. have found that PLA/wood with acetylated or heat-treated wood reduces water sorption compared to untreated PLA/wood [34]. Therefore, these works have shown that materials with PLA/wood have a high affinity for water.

In certain sectors such as the manufacture of air handling equipment, the adsorption of moisture by PLA/wood can be considered as a property of interest, and the mechanical properties are not a limiting factor. Authors’ studies show the promising potential of 3D printing and green desiccant materials for manufacturing ecological air dehumidification systems [35]. Using MEX and PLA/wood, it is possible to manufacture elements that allow air flows to be dehumidified in an efficient, eco-friendly, and low-cost way, compared to other conventional systems based on the use of vapour compression cycles [36].

The complex inter-relationship between process, structure, and properties in the context of MEX-manufactured biocomposites is still not fully understood. This results in a lower reliability of this technology in the context of biocomposites. The extrusion temperature of PLA/wood is slightly higher than that of pure PLA. Several authors have carried out tests to determine how the properties of the specimens change when they are printed at different temperatures between 195 °C and 300 °C [37]. Specimens printed at higher temperatures have lower porosity [38]. The recommended temperature for printing PLA/wood specimens is around 220 °C [39].

In this respect, the printing parameters can play a role in the sorption capacity of the printed PLA/wood. The layer height has influence on the amount of water absorbed when the printed specimen is immersed in water, showing a positive linear relationship between water absorption and layer height of the 3D printed samples [40]. However, the influence of this parameter on the water vapour adsorption capacity in saturated moist environment has not been studied. The relationship between the printing width and the absorption capacity of MEX printed specimens with PLA/PHA/wood filaments has been studied by Le Duigou et al. [41]; according to these authors, larger printing width is associated with higher adsorption capacity. On the other hand, Ang et al. [42] have studied the relationship between the raster width and the percentage of macropores in MEX printed structures that can serve as scaffolds for cell growth. They have concluded that lower raster width values are linked to higher porosity percentages.

There are authors who have investigated the impact of infill density on water purification filters made with PLA–carbon black fibres using MEX. Lagalante et al. have experimentally proven that filters made with a larger infill pattern (smaller channels) have a higher adsorption capacity of volatile organic compounds [43]. In turn, Zhang et al. have printed filters capable of retaining heavy metals and experimentally proved that infill patterns with a larger relative surface area per gram of printed material improve the retention efficiency of heavy metals [44].

In the context of PLA/wood 3D printing materials, several authors have explored different applications and behaviours of these composites. Le Duigou et al. have studied the dynamic hygromorphic behaviour of PLA/wood composites printed using FDM, focusing on a 4D printing application [41], since the material can evolve over time in response to an external stimulus. Krapez Tomec et al. have investigated the application of PLA/wood materials printed via FDM as 3D-printed shape-changing actuators [45]. Despite the promising applications of PLA/wood composites, there is a notable lack of research on the relationship between printing parameters and the actuation properties induced by natural-fibre composites, especially in the context of desiccant materials for air dehumidification.

This study addresses a significant gap in the literature by systematically investigating the primary water absorption mechanisms in PLA/wood composites with varying pine wood content, focusing on how nozzle diameter, layer height, and extrusion temperature influence porosity, surface roughness, and water vapor adsorption capacity. In contrast to previous studies, which aimed to reduce the water absorption capacity of PLA/wood composites through the use of coatings, heat treatments, or additives, the present work explores how to enhance this capacity for use in dehumidification applications. This approach turns what has traditionally been viewed as a limitation of the material into an advantage, offering a green and sustainable solution for the production of desiccant systems. This provides critical insights for optimising eco-friendly desiccant materials in air dehumidification systems.

## 2. Materials and Methods

In this work, various methods to improve water vapour adsorption in dehumidification elements manufactured via MEX by manipulating printing parameters were explored. First, three commercial PLA/wood filaments with different wood percentages were studied. For each material, water immersion tests were carried out to evaluate the relationship between the amount of wood and its water sorption capacity. Once the material with the highest water sorption was selected, an analysis was conducted on how several additive manufacturing parameters influence porosity, roughness, and water vapour adsorption capacity. Finally, three prototypes of water vapour adsorption systems were fabricated using the printing parameter values that provided the highest adsorption capacity and varying infill density. The infill density was used to generate air passage channels.

### 2.1. Material Evaluation

#### 2.1.1. Absorption Tests

Three filaments composed of PLA/wood with different percentages of wood were analysed in the present work. These percentages were chosen based on previous studies indicating that they offer a balance between mechanical stability, ease of processing in additive manufacturing technologies, and moisture adsorption capacity. Exceeding 25% wood content in a filament often poses challenges, as it negatively affects both printability and material strength, making it too fragile for functional applications [33]. The materials were composed of 5 wt.% pine wood (S5), 15 wt.% pine wood (S15), and 25 wt.% pine wood (S25); see Table 1.

**Figure 2:**
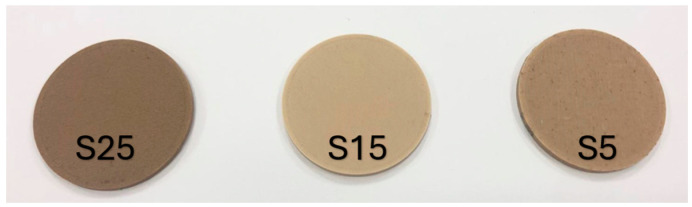
Specimens manufactured for the water absorption tests: 25 wt.% wood (S25), 15 wt.% wood (S15), 5 wt.% wood (S5).

The water sorption capacity was evaluated for the three materials following the ISO 62 standard [46]. For this analysis, three disc-shaped specimens of each material were manufactured. The dimensions of the specimens were 3.2 mm thick and 50.8 mm in diameter. An image with a specimen of each material is shown in Figure 2. The specimens were manufactured with a Creality Ender 3 printer (Shenzhen Creality 3D Technology Company, Shenzhen, China). The printing parameters used to obtain the specimens are shown in Table 2.

The testing process began by drying the specimens at 50 °C for 24 h, and then the dried specimens were weighed. The specimens were immersed in distilled water at 23 °C for 24 h. During the first 3 h, the specimens were weighed every 30 min after removing the surface water to measure the adsorbed water over time, and finally these were weighed after 24 h. The water sorption capacity of each material was obtained with the percentage of retained water, calculated with Equation (1).

(1) $$∆m=\frac{m_{f}-m_{i}}{m_{i}}\cdot 100$$

The diffusion (*D*), solubility (*S*), and permeability (*PM*) parameters were also obtained for each experimental test. *D* is one of the main mechanisms of water absorption of polymer composites with organic materials. This parameter was calculated according to Equation (2), where *b* is the thickness of the specimen, *θ* is the initial slope of the plot of *M(t)* vs. *t^1/2^*, and *M_m_* is the maximum gained weight of the specimen. *S* is related to the mass gain of water, as shown in Equation (3), where *m_w_* is the mass of water adsorbed at equilibrium state and *m_in_* is the initial mass of the material. *PM* is defined as the product of *D* and *S*; see Equation (4).

(2) $$D=\pi \cdot {\left[\frac{b\cdot \theta }{4\cdot M_{m}}\right]}^{2}$$

(3) $$S=\frac{m_{w}}{m_{in}}$$

(4) PM=D·S

#### 2.1.2. Fourier Transform Infrared Spectral Analysis (FTIR)

The infrared spectroscopy analysis of the different composites was recorded using a FT-MIR spectrophotometer with a Bruke Tensor 27 microscope (Bruker Optik GmbH, Ettlingen, Germany). The samples were formed in a potassium bromide matrix, and the scanning range was between 400 cm^−1^ and 4000 cm^−1^ with a resolution of 4 cm^−1^.

### 2.2. Evaluation of Printing Parameters

#### 2.2.1. Design of Experiments

Once the material was selected, the influence of the printing parameters on the porosity and water vapour adsorption capacity was studied. A Taguchi L8-type Design of Experiments (DOE) was carried out for this purpose. The input variables included in the DOE were extrusion temperature, layer height, and nozzle diameter. These variables were chosen based on information from the literature [47]. The extrusion temperature (T) values were varied from 210 °C to 230 °C, the nozzle diameter (D) values from 0.4 mm to 1.0 mm, and the layer height (LH) values from 0.1 mm to 0.3 mm. The combinations of the input variables used during the printing of the specimens are summarised in Table 3. The remaining printing parameters were kept constant; see Table 4.

**Figure 3:**
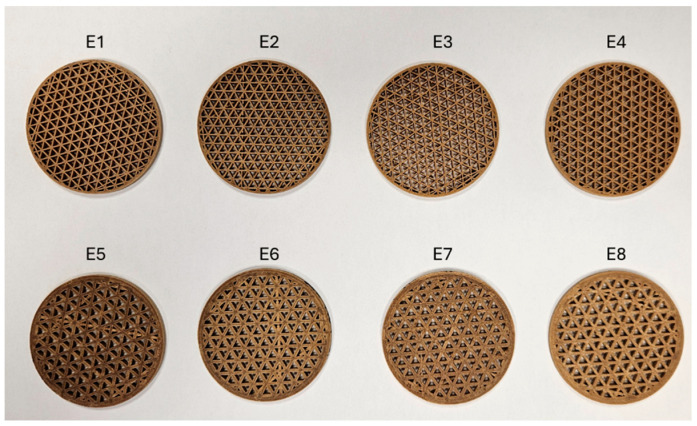
Specimens of PLA/wood printed according to the design of experiment.

In this analysis, the specimens were also designed and manufactured in the shape of a disc, but an infill density of 45% was used to generate greater contact surface area between the water vapour molecules and the material. The configuration of the printing process was performed using CURA software v. 5.2.1 [48]. For each specimen, a G-code file was generated, using the values indicated by DOE. As previously mentioned, a Creality Ender 3 printer was used. An image of the specimens manufactured according to the DOE is shown in Figure 3. The experimental results allowed obtaining analysis of variance (ANOVA) of porosity and water vapour adsorption capacity, determining the most influential input variables on these response variables, as well as generating main effects diagrams. The results of the DOE were processed in Minitab software v. 19.2 [49].

#### 2.2.2. Porosity

The porosity of each specimen was determined geometrically, assuming a cylindrical geometry. From the bulk density (*ρ_app_*), and knowing the theoretical density (*ρ_theo_*) of the filament, the relative densities (*ρ_rel_*) and total porosities (*P_Total_*) of the specimens were obtained using Equation (5) and Equation (6), respectively.

(5) $$\rho_{rel}=\frac{\rho_{app}}{\rho_{theo}}$$

(6) $$P_{Total}=\left(1-\rho_{rel}\right)\cdot 100$$

The total porosity values obtained included the values of closed porosity and open porosity of different ranges or sizes, so it can be represented according to Equation (7).

(7) $$P_{Total}=P_{closed}+P_{micro-meso}+P_{macro}+P_{supra\;macro}$$

where $P_{closed}$ is the closed porosity (obtained by helium pycnometry), $P_{micro-meso}$ corresponds to micro and mesoporosity (pore size < 50 nm, determined by N_2_ adsorption–desorption); $P_{macro}$ is the macroporous porosity (pores in the range from 50 nm to 100 μm determined by Hg intrusion porosimetry); and $P_{supra\;macro}$ corresponds to porosity higher than 100 μm.

#### 2.2.3. Water Vapour Adsorption

Moisture dehumidification systems are not immersed in water but work under moist air conditions. Therefore, experimental tests to evaluate the water vapour adsorption capacity under a saturated humid environment (relative humidity of 100%) were carried out. First, the specimens were placed in an oven at 50 ± 3 °C for 24 h, cooled in a desiccator, and immediately weighed on a balance (accuracy of 0.001 g). Subsequently, they were placed for 24 h in a closed chamber with a relative humidity equal to 100%. After 24 h, the surface water was removed from the specimens with a dry cloth, and they were weighed again on the balance. The amount of moisture adsorbed by the specimens was also calculated with Equation (1).

### 2.3. Surface Analysis

**Figure 4:**
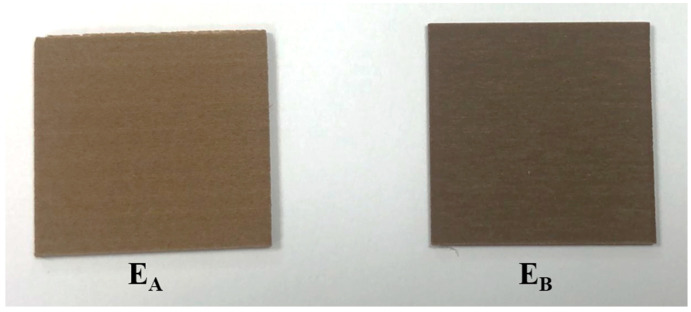
Specimens to analyse the surface of the materials with the highest (E_A_) and lowest (E_B_) water vapour adsorption capacity.

Surface quality is assessed using the surface area roughness parameter S_a_ (arithmetical mean height), as defined by ISO 25178 [50], in order to verify the relationship between water vapour adsorption and surface roughness. This parameter represents the arithmetic mean of the absolute height values relative to the surface’s mean plane. Commonly, the average arithmetic deviation R_a_ is used to characterise the surface texture along a section of the machined part. Among various roughness parameters, R_a_ is valued for providing extensive information about the future operational performance of the surface it characterises. When evaluating the roughness of a particular surface, the S_a_ parameter holds similar significance and relevance to the R_a_ parameter used for surface profile roughness in sectional analysis. Two specimens were manufactured with printing parameters that allowed the highest and lowest water vapour adsorption capacity, the E_A_ and E_B_ samples, respectively, with dimensions of 39 × 39 × 2 mm, as shown in Figure 4. Roughness and primary surface were measured by means of a LEICA model DCM8 confocal microscope and scanning electron microscopy (SEM).

Additionally, the relationship of the printing parameters on the moisture adsorption capacity was evaluated using water vapour adsorption–desorption isotherms to find the amount of moisture adsorbed by each material under different relative pressures. These tests were carried out at 298 K. A Micromeritics 3Flex Surface Characterization system (Micromeritics, Lincoln, UK) was used to obtain these sorption isotherms.

### 2.4. Evaluation of Infill Density on Moisture Removal Capacity

**Figure 5:**
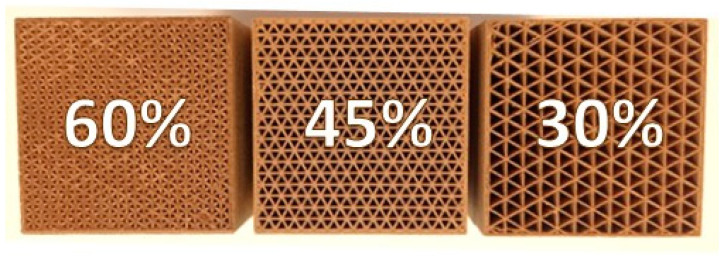
Desiccant prototypes printed on PLA/wood via MEX with different infill density.

Three prototypes of water vapour adsorption systems were manufactured using the printing parameters that, according to the adsorption tests in the previous stage, provided the highest adsorption capacity. Each prototype was developed by varying the infill density value: 30%, 45%, 60%; see Figure 5. The infill density was used to generate air passage channels. The objective of this analysis was to evaluate how the infill density, and thus the size of the channels, influences the adsorption performance when a humid air flow circulates over its surface. The three prototypes had external dimensions of 50 × 50 × 150 mm (height × width × length).

**Figure 6:**
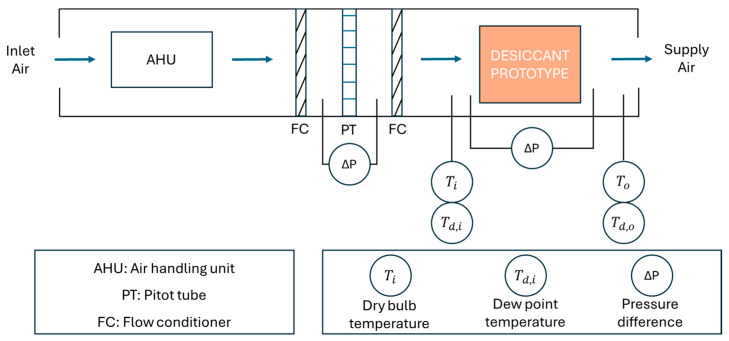
Schematic description of the experimental setup.

The three manufactured water vapour adsorption systems were tested in an air handling laboratory (Figure 6). The technical characteristics of the experimental setup were shown in previous work [35]. This experimental setup was designed to generate different values of temperature, humidity, and air flow rate. Several sensors of dry bulb temperature and dew point temperature were located at the inlet and outlet of the prototypes. In this study, dynamic experimental tests were carried out, setting the inlet air conditions at a dry bulb temperature of 32 °C, an air humidity ratio of 15 g/kg, and an air flow rate of 40 m^3^/h. For each experimental test, the moisture adsorption capacity was determined according to Equation (8), where ω_i_ and ω_o_ are inlet and outlet air humidity ratio values, respectively.

(8) $$\Delta \omega =\omega_{i}-\omega_{o}$$

## 3. Results

### 3.1. Water Absorption Capacity and Chemical Composition of PLA/Wood Composites

**Figure 7:**
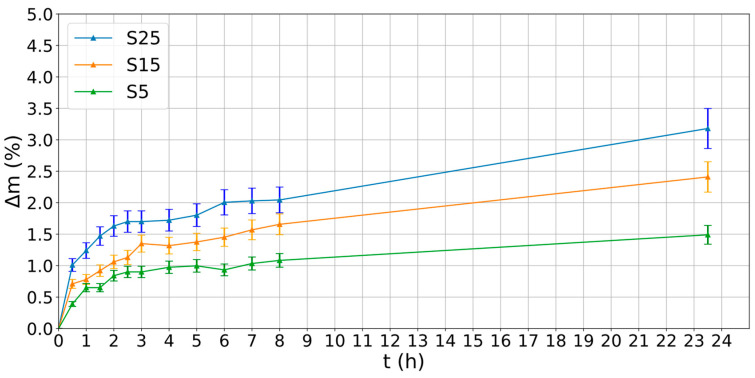
Water absorption capacity of the three PLA/wood composites.

The water adsorption for the three selected materials composed of PLA and wood was analysed. The results of the absorption capacity tests are shown in Figure 7. The S25 material was the one that reached the highest Δ*m*, up to a value of 3.2% at 24 h. The S25 curve also showed that the absorption capacity was faster. However, the material with the lowest Δ*m* values was S5, with a maximum value of 1.5% at 24 h. This suggests that the greater the amount of wood contained in the composite, the greater the absorption capacity. These results are in agreement with those obtained by other authors in previous work [25].

The water absorption results allowed the diffusion coefficient (*D*), thermodynamic solubility (*S*), and permeability (*PM*) to be obtained for each material. The results of *D*, *S*, and *PM* for the three materials are shown in Table 5. These results showed that water molecules diffuse more easily in filaments with higher pine wood content (S25), that is, the diffusion process was faster. In addition, S25 was the material that had the highest values of solubility and permeability to water. The main factors affecting the water absorption of wood composites were the following: fibre volume fraction, fibre orientation, exposed surface area and temperature. PLA has less affinity for water than wood, so the amount of wood was mainly responsible for absorbable water [28]. Therefore, based on the results obtained, the S25 material was selected to evaluate the printing parameters, as it exhibited the highest values of water absorption capacity, diffusion coefficient, solubility, and permeability.

**Figure 8:**
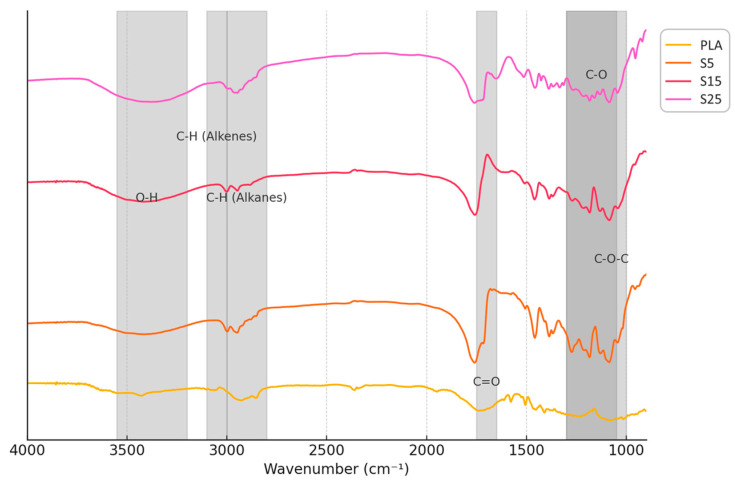
FTIR spectroscopic analysis of the different samples developed.

The FTIR spectra of pure PLA and PLA/wood composites (S5, S15, S25) are shown in Figure 8. The distinct absorption bands characteristic of their respective chemical compositions can be appreciated. In the region between 3000 cm^−1^ and 3600 cm^−1^, a broad absorption band is observed, corresponding to O–H stretching vibrations. This band is associated with hydroxyl groups from water and cellulose. As the wood content increases in the samples (S5, S15, and S25), the intensity of this band grows, reflecting the increasing presence of lignocellulosic materials, particularly cellulose and water adsorbed onto the wood fibres.

Around 2800 cm^−1^–3100 cm^−1^, the C–H stretching bands characteristic of alkanes are present in all samples. This band is associated with both PLA and the cellulose/hemicellulose components of wood. The increased intensity in the wood composites can be attributed to the contribution of lignocellulosic components, as confirmed by previous studies [51]. These C–H stretches are particularly relevant for identifying the polysaccharide structure of the wood.

In the region between 1700 and 1800 cm^−1^, a strong absorption band is observed for all samples, which corresponds to the C=O stretching vibrations of ester groups in PLA. This peak, however, decreases in intensity as the wood content increases, likely due to the dilution of PLA by wood particles. This region is also associated with carbonyl groups from hemicellulose and lignin, which may further contribute to the spectral features of the composite samples.

At approximately 1600–1610 cm^−1^, a minor absorption peak is present in the wood-containing samples, corresponding to O–H hydroxyl and C=O stretching vibrations. This peak is characteristic of lignin and water, as seen in the S15 and S25 spectra. The absence of significant intensity in the PLA sample supports the hypothesis that these signals arise from the wood components.

The region between 1455 and 1370 cm^−1^ shows increased absorption in the wood composites, which can be attributed to C–H bending vibrations associated with cellulose, hemicellulose, and lignin. The intensity of these bands correlates with the wood content, particularly in the S15 and S25 samples, confirming the presence of polysaccharides within the composites. These bands are not as prominent in the PLA spectrum, indicating the absence of such lignocellulosic material.

Significant absorption is observed between 1250 and 1150 cm^−1^, which corresponds to C–O–C stretching vibrations. This region is characteristic of lignin and hemicellulose and shows a clear increase in intensity as the wood content rises. While PLA also exhibits C–O–C stretching due to its polyester structure, the increased intensity in this region in S15 and S25 indicates the additional contribution from wood components.

Lastly, the absorption peak around 1030 cm^−1^ is notably more intense in the wood composites, particularly in S15 and S25, and is associated with C–O stretching vibrations of lignin. The PLA sample shows a much weaker absorption in this region, highlighting the distinct contribution of the lignocellulosic matrix in the wood composites. These results validate the composition of the material used in the water adsorption tests.

### 3.2. Analysis of the Influence of Printing Parameters

The relationship between porosity and water vapour adsorption capacity for different manufacturing parameters was analysed for the S25 material.

#### 3.2.1. Analysis of Porosity

**Figure 9:**
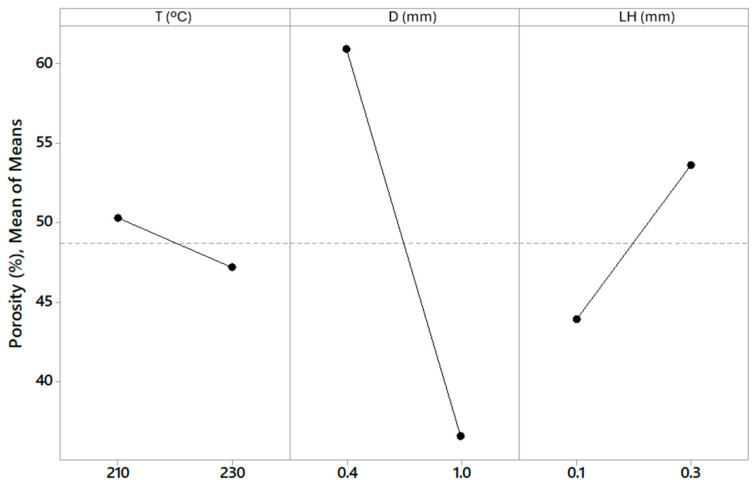
Main effects plot of T, D, and LH for porosity.

The total porosity values obtained for each specimen are shown in Table 6. The specimens with the highest porosity were E1, E3, and E4, while E5 and E6 were the specimens with the lowest porosity. The main effects plot for porosity is shown in Figure 9. There was a tendency to generate higher porosity with smaller nozzle diameter in the studied range of 0.4 mm to 1 mm. Increasing the layer height from 0.1 mm to 0.3 mm, higher porosity was also observed, while the temperature had a minor effect on porosity, although a slight tendency for porosity to decrease with increasing temperature could be seen.

These results have allowed an analysis of variance (ANOVA) to be carried out (Table 7). The diameter of the nozzle was the most influential parameter (*p*-value = 0.001). The porosity was also influenced by the layer height (*p*-value = 0.007), although to a minor degree (Figure 8). Temperature had no significant influence on adsorption capacity. These results indicated that the lowest porosity obtained in the manufacturing process is for the largest nozzle diameter (1.0 mm) and the smallest layer height (0.1 mm), while the smallest nozzle diameter (0.4 mm) and the largest layer height (0.3 mm) generated the highest porosity in the manufacturing process with MEX AM in the range studied. The relation of porosity and nozzle diameter and layer height were consistent with those obtained with Fischer et al. [47].

#### 3.2.2. Evaluation of Adsorption Capacity

**Figure 10:**
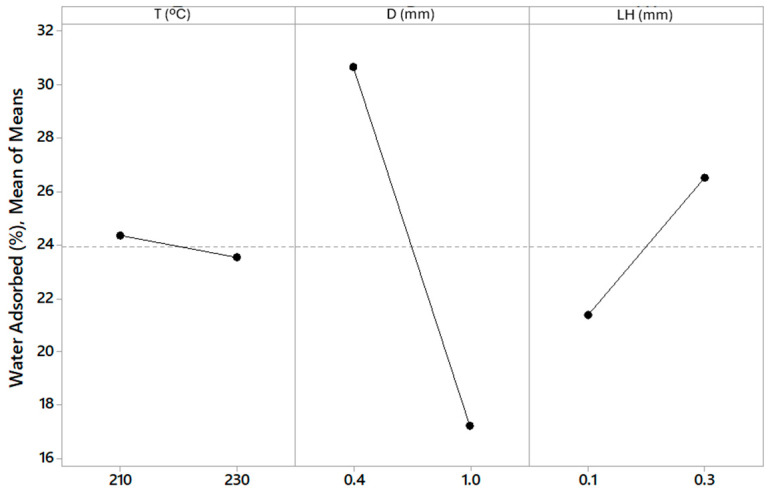
Main effects plot of T, D, and LH for capacity of adsorption.

The percentages of water vapour adsorption by each of the printed specimens during the test carried out in a saturated humid environment are shown in Table 8. The specimens E1, E3, and E4 adsorbed the greatest amount of water vapour, while E5 and E6 adsorbed the least amount. From these values, the main effects plot is shown in Figure 10. A tendency to adsorb more water vapour was observed with smaller nozzle diameter in the studied range of 0.4 mm to 1 mm. An increase in water vapor adsorption was also observed as the layer height increased from 0.1 mm to 0.3 mm. Temperature variation showed no significant trend in water vapour adsorption.

A statistical analysis using ANOVA was conducted to determine the significance of the observed differences in water adsorption capacity with respect to process parameters such as nozzle diameter and layer height (Table 9). The *p*-values obtained from the variance analysis were included to validate the influence of printing parameters on moisture adsorption capacity. A *p*-value below 0.05 was considered statistically significant. Printing parameters that had the most influence on the adsorption capacity of specimens when they were introduced in a saturated humid environment were the nozzle diameter (*p*-value = 0.002) and the layer height (*p*-value = 0.047). Therefore, these results indicated that the lowest water vapour adsorption capacity obtained in the manufacturing process was for the largest nozzle diameter (1.0 mm) and the smallest layer height (0.1 mm), while for the smallest nozzle diameter (0.4 mm) and the largest layer height (0.3 mm), the highest water vapour adsorption capacity was achieved in the manufacturing process with MEX AM in the range studied.

**Figure 11:**
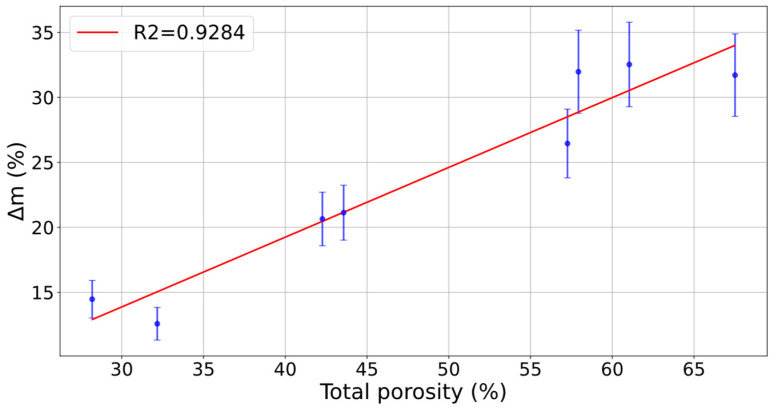
Correlation between total porosity and water vapour adsorption capacity.

The relationship between porosity and adsorption capacity is shown in Figure 11. The relationship between both variables follows a linear trend, so that the higher the porosity, the higher the water vapour adsorption. The correlation obtained has an R^2^ value of 0.9284. The adsorption capacity of each specimen is clearly correlated to its porosity (Figure 11). The desiccant elements with higher porosity were those with higher adsorption capacity, that is, E1, E3, and E4 were the specimens with the highest values of porosity and consequently adsorption capacity. The porosity of the printed samples was determined using geometric methods and evaluated based on printing parameters (nozzle diameter, layer height, extrusion temperature). A direct relationship was observed between increased porosity and both larger layer heights and smaller nozzle diameters. This increase in porosity significantly contributed to the enhancement of water adsorption capacity, as higher porosity provides a greater surface area for interaction with water molecules. These results are consistent with findings from other authors [41,42], who have demonstrated that optimising printing parameters can increase the porosity of printed materials to enhance their functional capacity. Optimising porosity and these parameters is key for applications requiring high-performance moisture adsorption [52].

### 3.3. Analysis of Roughness and Primary Surface

**Figure 12:**
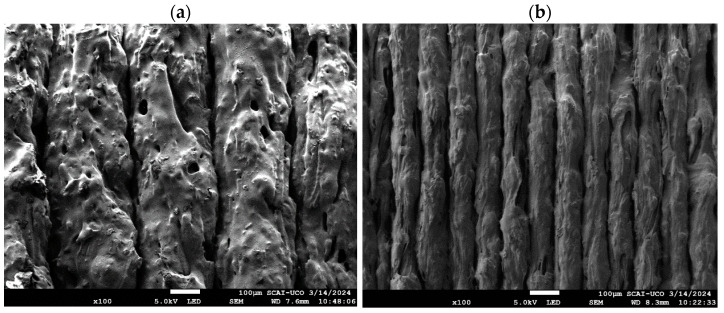
SEM images of samples E_A_ (**a**) and E_B_ (**b**).

The surfaces of the specimens with the printing parameters that allowed the highest and lowest water vapour adsorption capacity were analysed. The manufacturing parameters with the highest adsorption capacity were D = 0.4 mm, LH = 0.3 mm, and T = 210 °C (E_A_ sample), and those with the least were D = 1.0 mm, LH = 0.1 mm, and T = 230 °C (E_B_ sample). Images of both samples, made with a scanning electron microscope (SEM), are shown in Figure 12. It can be observed that a greater concentration of holes and deformities was obtained in the E_A_ sample compared to the E_B_ sample. The diameter of the filament generated in the E_A_ sample was also much larger than that of the E_B_ sample, because the layer height was higher in E_A_ than in E_B._

**Figure 13:**
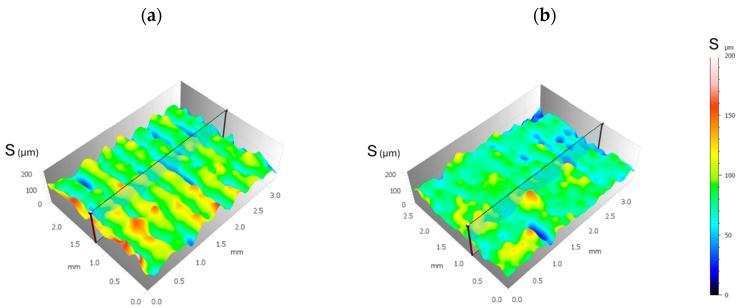
Surface profiles of the samples (**a**) E_A_ and (**b**) E_B_.

Profiles of the primary surface and roughness of these two specimens were obtained with a confocal microscope; see Figure 13. Significant differences are seen in the surface of the E_A_ sample with respect to that of E_B_. The E_A_ sample exhibited more pronounced peaks and valleys, showing a rougher texture. The configuration of the E_A_ sample contributed to this roughness, as each deposited layer was thicker. In contrast, the E_B_ sample exhibited a smoother texture with less pronounced height variations, largely influenced by the smaller layer height. The results of roughness (R_a_) and primary surface (S_a_) are also shown in Table 10. The values of R_a_ and S_a_ for the E_A_ sample were 40% and 35% higher, respectively, compared to the E_B_ sample, indicating a higher surface roughness of sample E_A_. Previous work has also shown that manufacturing parameters modify the roughness and primary surface [53,54].

**Figure 14:**
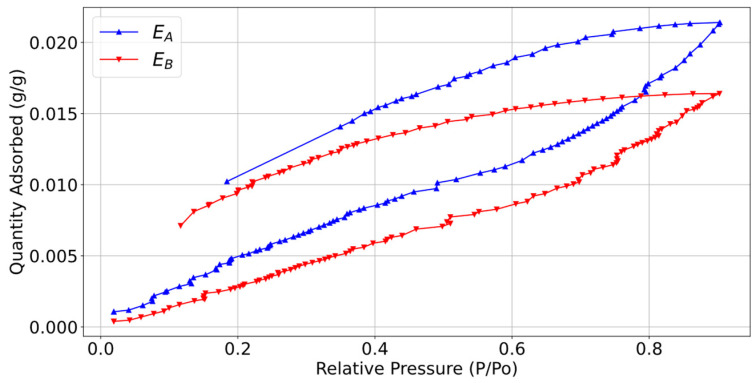
Adsorption–desorption isotherms for the samples E_A_ and E_B_.

Adsorption–desorption isotherms were carried out for samples E_A_ and E_B_, as shown in Figure 14. The trends of the adsorption–desorption curves were similar for both printing parameter settings, with a visible hysteresis occurring. This suggests that strong bonds were formed between the material and the water molecules. The maximum adsorption values for E_A_ and E_B_ were 1.2 mmol/g and 0.9 mmol/g, respectively, at a relative pressure of 0.9. Therefore, the SA sample, with its greater roughness and primary surface area, adsorbed 25% more water than the SB sample.

### 3.4. Impact of Infill Density in Capacity of Adsorption

**Figure 15:**
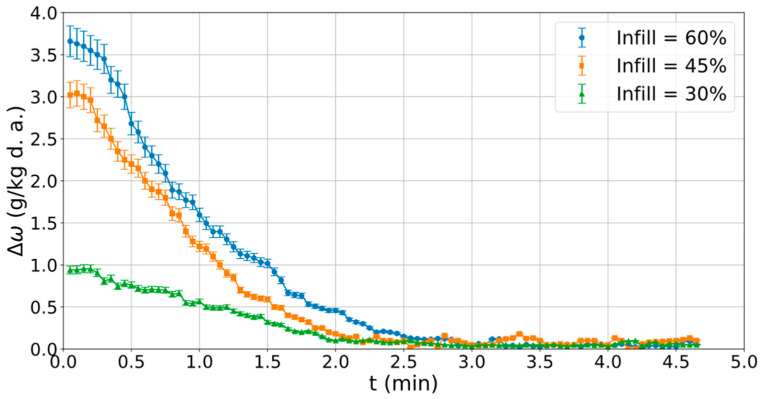
Differences in humidity ratio (Δω) between the inlet and outlet airflow for the three prototypes.

Three prototypes of a desiccant system with different infill density (30%, 45% and 60%) were printed and experimentally tested in an air conditioning laboratory to analyse their water vapour adsorption capacity. The infill density was used to generate air passage channels. A humid air flow of 40 m^3^/h was circulated through the prototypes. The inlet air temperature and inlet air humidity ratio were 35 °C and 15 g/kg, respectively. The results of the difference in humidity ratio (Δω) between the inlet and outlet airflow for the three prototypes are shown in Figure 15. It can be observed than the desiccant prototype with the infill density of 60%, i.e., with the smallest channel size, had the highest moisture adsorption capacity over time. Following this was the prototype with the infill density of 45%, and finally, the prototype with a filling density of 30%, i.e., with the largest channel size, had the least moisture adsorption capacity. Therefore, the mass transfer from air to the desiccant material was greater when the size of the channels was smaller, increasing the moisture adsorption capacity.

The maximum Δω values were reached at the beginning of the dehumidification process, 3.7 g/kg, 3 g/kg, and 1 g/kg for the prototypes with filling densites of 60%, 45%, and 30%, respectively, as shown in Figure 15, due to the water vapour pressure difference between the humid air flow and the dry material. The increase in moisture adsorption when raising the infill density from 30% to 45% was noticeable. However, from 45% to 60%, the increase was much smaller. Therefore, an infill density between 45% and 60% may be optimal for improved water vapour adsorption. These Δω values decreased over time as the material retained moisture, and therefore, the difference in water vapour between the air and the material was reduced. All prototypes were saturated with moisture after 2.5 min, as shown in Figure 15, so Δω was close to 0 g/kg. These trends agreed with those obtained in previous works on desiccant dehumidification systems [35]. These results show the importance of adjusting the fill density to increase the desiccant capacity of the system.

PLA/wood composites exhibited lower moisture adsorption capacity compared to traditional desiccant materials such as silica gel [55]. However, they offer significant advantages, including lower production costs and the ability to be fabricated via 3D printing, providing greater flexibility in design and application [4]. Furthermore, these materials are biodegradable, which aligns with the current trend towards more sustainable materials in the industry [22]. Comparatively, PLA/wood composites demonstrate similar adsorption capacities to other eco-friendly desiccant materials made from polymers and agricultural waste. For example, Robledo-Ortiz et al. [56] showed that polyethylene/agave composites achieved water adsorption of 11.7% to 20%. These findings indicate that PLA/wood composites are competitive with other bio-based materials, offering the added advantage of customisable 3D printing capabilities.

## 4. Conclusions

This study explored the potential of using PLA/wood composites to produce desiccant elements for air dehumidification via extrusion-based additive manufacturing. The research focused on evaluating the influence of printing parameters on water vapour adsorption and understanding the primary mechanisms of water absorption for materials with varying pine wood content. The main conclusions of this experimental work were:

- The investigation into the water absorption capacity of PLA/wood composites confirmed a direct correlation with pine wood content. The material with the highest wood content (S25) showed superior performance in water absorption, diffusion coefficient, solubility, and permeability compared to those with lower wood content (S15, S5). Thus, S25 was identified as optimal for further analysis of printing parameters, validating the hypothesis that higher wood content significantly enhances water absorption capacity, making it more effective for moisture management applications.
- Analysis of printing parameters revealed the importance of nozzle diameter and layer height in influencing water vapor adsorption. A smaller nozzle diameter and larger layer height increased porosity and surface roughness, leading to higher water vapor adsorption. On the other hand, extrusion temperature had minimal impact within the studied range.
- The impact of infill density on water vapor adsorption was evident, with higher densities resulting in greater adsorption. Although the 60% infill density yielded the best results, the 45% density achieved similar outcomes. This finding has both technological and economic significance, as the use of lower densities is associated with reduced printing times and material savings.

These findings provide valuable insights into how material composition and printing parameters affect the performance of PLA/wood composites in moisture adsorption applications. While this study focused on optimising the printing parameters to enhance moisture adsorption capacity, the biodegradability of the PLA/wood composite material is a crucial characteristic for sustainable applications. The evaluation of the longevity and biodegradability of both the filament and the printed parts will be addressed in future studies, providing a more complete perspective on their environmental impact. Additionally, these insights could be utilised to manufacture efficient desiccant dehumidification systems, such as desiccant wheels, and to study how temperature and humidity of the air influence their adsorption capacity.

## Figures and Tables

**Table 1:** Density and percent of pine wood in composites of S5, S15, and S25.

Material	Density (g/cm^3^)	Percent of Wood (wt.%)	Percent of PLA (wt.%)
S25	1.0119	25	75
S15	1.0952	15	85
S5	1.2012	5	95

**Table 2:** Printing parameters used to manufacture the specimens for absorption test.

Parameter	Value
Infill Pattern	Line
Infill Density (%)	100
Printing Speed (mm/s)	50
Printing Acceleration (mm/s^2^)	500
Flow (%)	100
Build Plate Temperature (°C)	50

**Table 3:** DOE (Taguchi L8) used in the work.

Specimen	Extrusion Temperature, T (°C)	Nozzle Diameter, D (mm)	Layer Height, LH (mm)
E1	210.0	0.4	0.1
E2	230.0	0.4	0.1
E3	210.0	0.4	0.3
E4	230.0	0.4	0.3
E5	210.0	1.0	0.1
E6	230.0	1.0	0.1
E7	210.0	1.0	0.3
E8	230.0	1.0	0.3

**Table 4:** Constant printing parameters of the manufacturing process.

Parameter	Value
Infill Pattern	Triangle
Infill Density (%)	45
Printing Speed (mm/s)	50
Printing Acceleration (mm/s^2^)	500
Flow (%)	100
Build Plate Temperature (°C)	50

**Table 5:** Results of diffusion coefficient, solubility, and permeability for the three materials.

Material	*D* (m^2^/s)	*S* (kg/kg)	*PM* (m^2^/s)
S25	3.70 × 10^−7^	0.031	1.15 × 10^−8^
S15	3.182 × 10^−7^	0.024	7.67 × 10^−9^
S5	2.512 × 10^−7^	0.014	3.57 × 10^−9^

**Table 6:** Total porosity calculated for each specimen, in percentage.

Specimen	m_i_ (g)	V (cm^3^)	ρ_app_ (g/cm^3^)	ρ_rel_ (g/cm^3^)	P_T_ (%)
E1	2.69	5.87	0.46	0.42	57.93
E2	2.76	5.92	0.47	0.43	57.26
E3	2.06	5.82	0.35	0.32	67.51
E4	2.52	5.93	0.42	0.39	61.04
E5	4.56	6.17	0.74	0.68	32.17
E6	4.76	6.08	0.78	0.72	28.19
E7	3.91	6.36	0.62	0.56	43.57
E8	3.77	5.99	0.63	0.58	42.27

**Table 7:** Analysis of variance (ANOVA) of T, D, and LH for porosity.

Source	DOF	Seq SS	Contribution	Adj SS	Adj MS	F	*p*-Value
T	1	19.31	1.35	19.31	19.31	2.67	0.178
D	1	1189.35	83.40	1189.35	1189.35	164.28	0.000
LH	1	188.54	13.22	188.54	188.54	26.04	0.007
Error	4	28.96	2.03	28.96	7.24		
Total	7	1426.16	100.00				

**Table 8:** Water vapour adsorbed by the specimens.

Specimen	Δm (%)
E1	31.97
E2	26.45
E3	31.71
E4	32.53
E5	12.58
E6	14.47
E7	21.13
E8	20.64

**Table 9:** Analysis of variance (ANOVA) of T, D, and LH for adsorption capacity.

Source	DOF	Seq SS	Contribution	Adj SS	Adj MS	F	*p*-Value
T	1	1.361	0.31	1.361	1.361	0.21	0.671
D	1	362.343	81.89	362.343	362.343	55.71	0.002
LH	1	52.736	11.92	52.736	52.736	8.11	0.047
Error	4	26.017	5.88	26.017	6.504		
Total	4	442.458	100.00				

**Table 10:** Surface roughness parameters for each specimen.

	**Parameters**	**E_A_**	**E_B_**
Primary surface	Sa [μm]	16.45	12.20
Rugosity	Ra [μm]	11.28	8.05

## Data Availability

The original contributions presented in the study are included in the article, further inquiries can be directed to the corresponding author.
